# *N*‐glycan signatures identified in tumor interstitial fluid and serum of breast cancer patients: association with tumor biology and clinical outcome

**DOI:** 10.1002/1878-0261.12312

**Published:** 2018-05-14

**Authors:** Thilde Terkelsen, Vilde D. Haakensen, Radka Saldova, Pavel Gromov, Merete Kjær Hansen, Henning Stöckmann, Ole Christian Lingjærde, Anne‐Lise Børresen‐Dale, Elena Papaleo, Åslaug Helland, Pauline M. Rudd, Irina Gromova

**Affiliations:** ^1^ Computational Biology Laboratory Danish Cancer Society Research Center Copenhagen Denmark; ^2^ Department of Cancer Genetics Institute for Cancer Research The Norwegian Radium Hospital, Oslo University Hospital Norway; ^3^ NIBRT GlycoScience Group National Institute for Bioprocessing Research and Training Dublin 4 Ireland; ^4^ Danish Cancer Society Research Center Genome Integrity Unit Breast Cancer Biology Group Copenhagen Denmark; ^5^ Present address: AbbVie Inc. 1 North Waukegan Road North Chicago IL 60064 USA

**Keywords:** biomarker, blood, heterogeneity, *N*‐glycan, tumor infiltrating lymphocytes, tumor microenvironment

## Abstract

Particular *N*‐glycan structures are known to be associated with breast malignancies by coordinating various regulatory events within the tumor and corresponding microenvironment, thus implying that *N*‐glycan patterns may be used for cancer stratification and as predictive or prognostic biomarkers. However, the association between *N*‐glycans secreted by breast tumor and corresponding clinical relevance remain to be elucidated. We profiled *N*‐glycans by HILIC UPLC across a discovery dataset composed of tumor interstitial fluids (TIF,* n* = 85), paired normal interstitial fluids (NIF,* n* = 54) and serum samples (*n* = 28) followed by independent evaluation, with the ultimate goal of identifying tumor‐related *N*‐glycan patterns in blood of patients with breast cancer. The segregation of *N*‐linked oligosaccharides revealed 33 compositions, which exhibited differential abundances between TIF and NIF. TIFs were depleted of bisecting *N*‐glycans, which are known to play essential roles in tumor suppression. An increased level of simple high mannose *N*‐glycans in TIF strongly correlated with the presence of tumor infiltrating lymphocytes within tumor. At the same time, a low level of highly complex *N*‐glycans in TIF inversely correlated with the presence of infiltrating lymphocytes within tumor. Survival analysis showed that patients exhibiting increased TIF abundance of GP24 had better outcomes, whereas low levels of GP10, GP23, GP38, and coreF were associated with poor prognosis. Levels of GP1, GP8, GP9, GP14, GP23, GP28, GP37, GP38, and coreF were significantly correlated between TIF and paired serum samples. Cross‐validation analysis using an independent serum dataset supported the observed correlation between TIF and serum, for five of nine *N*‐glycan groups: GP8, GP9, GP14, GP23, and coreF. Collectively, our results imply that profiling of *N*‐glycans from proximal breast tumor fluids is a promising strategy for determining tumor‐derived glyco‐signature(s) in the blood. *N*‐glycans structures validated in our study may serve as novel biomarkers to improve the diagnostic and prognostic stratification of patients with breast cancer.

**Abbreviations**
BC: breast cancer
DAA: differential abundance analysis
ER: estrogen receptor
FFPE: formalin‐fixed paraffin‐embedded
GP: glycan peaks
HE: hematoxylin and eosin
HILIC: hydrophilic interaction liquid chromatography
IHC: immunohistochemistry
MDS: multidimensional scaling
NIF: normal interstitial fluids
PgR: progesterone receptor
SLex: Sialyl Lewis structure
TIF: tumor interstitial fluids
TILs: tumor infiltrating lymphocytes
TNBC: triple negative breast cancer
UPLC: ultra‐performance liquid chromatography

## 1 Introduction

Breast cancer (BC) is the most common cancer worldwide among women, with more than 1 300 000 new cases diagnosed every year. BC is the leading cause of cancer‐related deaths among women (Torre *et al*., 2016). Numerous studies have established that stepwise accumulation of multiple genetic and epigenetic alterations in epithelial cancer cells (Cancer Genome Atlas, 2012), as well as changes in stromal composition, drive and direct the progression of breast cancer (Beck *et al*., 2011). These studies highlight the heterogeneity and complexity of breast malignancies and point to a major challenge in the development of targeted therapeutics.

A growing body of evidence points to a crucial role of the multidirectional network communications between malignant epithelial cells and the tumor microenvironment in tumor evolution and progression. Multidirectional signaling events within the tumor stroma are implemented through the tumor interstitial fluid (TIF), which forms at the interface between circulating bodily (lymph and blood) and intracellular fluids. TIF facilitates the exchange of ions, proteins, cytokines, and miRNA within the interstitial space (Espinoza *et al*., 2016; Gromov *et al*., 2013; Papaleo *et al*., 2017). Various biomolecules are released by tumor and stromal cells into the interstitium (Horimoto *et al*., 2012; Zhang *et al*., 2017) and subsequently drain through the lymphatic system into the bloodstream, where they can be detected and quantified (Surinova *et al*., 2011). Given the high concentration of potential cancer‐specific biomolecules within the local tumor milieu (Ahn and Simpson, 2007), interstitial fluid is considered to be a valuable resource for BC biomarker discovery (Wagner and Wiig, 2015).

Glycosylation is a template‐free enzymatic process that produces glycosidic linkages of monosaccharides to macromolecules such as carbohydrates, lipids, and proteins through the sequential attachment of glycan moieties in a function‐specific context. This post‐translational modification is a well‐known hallmark of cancer (Pinho and Reis, 2015) and is implicated in almost all molecular and metabolic events in normal and malignant cells. These events include protein folding and stability, cell–cell interaction, angiogenesis, immune modulation, cell signaling, and gene expression (Moremen *et al*., 2012). Two major types of glycosylation (*N*‐linked and *O*‐linked) coexist in mammalian cells and often occur simultaneously on the same target macromolecules (Pinho and Reis, 2015).

The involvement of *N*‐glycosylation in the development and progression of BC has been documented by *in vitro* and *in vivo* studies (Julien *et al*., 2006). *N*‐glycan branching, particularly the increased expression of complex β‐1,6 branched *N‐*linked glycans, is often associated with more aggressive tumor behavior, such as enhanced migration, invasion, and metastatic potential (Contessa *et al*., 2008). In contrast, the expression of bisecting glycans strengthens cell adhesion and is associated with cancer suppression (Taniguchi and Kizuka, 2015). Several *N*‐glycan patterns with altered circulating glycan structures originating from either a primary tumor or from other organs in response to a neoplastic process have recently been described (Kyselova *et al*., 2008). Levels of biantennary *N*‐glycan chains as well as α‐2,3‐linked sialic acid‐modified *N*‐glycans are often decreased in sera of patients with BC, compared to healthy controls. The same tendency is observed in the sera of lung cancer patients, and it was suggested that an aberrant *N*‐glycan signature based on serum glycan analysis could be used to distinguish cancer types (Lan *et al*., 2016). However, no robust blood glycan markers for BC have been identified to date, mainly because of the high degree of complexity and dynamic range of biomolecules (>10 orders of magnitude) circulating in the bloodstream. Furthermore, similar types of molecules are externalized from other body tissues and organs under physiological conditions.

To identify tumor‐derived *N*‐glycans patterns, we investigated the secreted glycome by profiling *N*‐glycans released from matched tumors (TIF), normal mammary tissues (NIF), and serum samples using hydrophilic interaction liquid chromatography (HILIC) ultra‐performance liquid chromatography (UPLC) (Saldova *et al*., 2014). The aims of our study were (a) to compare *N*‐glycans secreted directly from tumor and stromal cells to correlate the *N*‐glycan profiles and the corresponding abundances in paired TIF and serum samples; (b) to explore whether the appearance of particular glycoforms in TIF is correlated with the presence of tumor infiltrating lymphocytes (TILs) in corresponding tumors; (c) to examine a potential association between *N*‐glycan levels and clinical outcome; and (d) to evaluate our data and results of analysis using an independent cohort of the normal, benign, and BC blood samples.

## 2 Materials and methods

### 2.1 Clinical samples: tumor tissue, matched nonmalignant tissue, and serum

Fresh tissue samples were collected from patients defined as high risk according to the Danish Breast Cooperative Group (DBCG; http://www.dbcg.dk accessed on October 22, 2009) who had undergone a mastectomy between 2003 and 2012 as part of the Danish Center for Translational Breast Cancer Research program (Gromov *et al*., 2014) at Copenhagen University Hospital. The criteria for high‐risk cancers, applied by the DBCG, are age below 35 years, and/or a tumor diameter of more than 20 mm, and/or a histological malignancy 2 or 3, and/or negative estrogen (ER) and progesterone (PgR) receptor statuses, and/or a positive axillary status. Mastectomy enables pathologist to dissect a tissue sample from a nonmalignant area located relatively distant to the tumor, that is, 5 cm. at least. We used such criteria for dissection of normal breast lesions to avoid any impact of cancer field cancerization, which has been observed in histologically normal breast biopsies located 1 cm from the tumor margins, but not in lesions resected 5 cm from the tumor or obtained from reduction mammoplasty (Heaphy *et al*., 2006; Trujillo *et al*., 2011). All normal tissue specimen dissected from the breast after mastectomy were morphologically and histologically evaluated (Russo and Russo, 2014) to ensure normal epithelial acini and ducts structures.

All the patients presented a unifocal tumor, and none of the patients had a history of breast surgery or had received preoperative treatment (naive samples). Patients were followed after surgery, and cancer‐specific survival was measured from the date of primary surgery until the date of death from BC. Death records were complete up to October 08, 2014 and served as the censoring date. Registered clinicopathological data for the patients were available from the Department of Pathology, Rigshospitalet, Copenhagen University Hospital, Denmark. This study was conducted in compliance with the Helsinki II Declaration, and written informed consent was obtained from all participants and approved by the Copenhagen and Frederiksberg regional division of the Danish National Committee on Biomedical Research Ethics (KF 01‐069/03).

At the time of collection, each tissue specimen was divided into two pieces. One piece was stored at −80 °C and was subsequently prepared as a formalin‐fixed paraffin‐embedded (FFPE) sample that was sectioned, mounted on glass slides, and stained for histological characterization, tumor subtyping, TIL scoring, and immunohistochemistry (IHC) analysis. The second biopsy piece was placed in PBS at 4 °C within 30–45 min of surgical excision and then was subjected to interstitial fluid recovery (see below).

Matched sera were obtained from women enrolled in the Danish Center for Translational Breast Cancer Research program who underwent surgery between 2001 and 2006. Blood samples were collected preoperatively following a standardized protocol (Wurtz *et al*., 2008). Briefly, serum was collected in serum‐separating tubes and was left on the bench for 30 min before centrifuging for 10 min at 2000 G.

The separate serum cohort, Mammographic Density and Genetis (MDG), consisted of serum *N*‐glycan profiles from 107 BC patients and 62 healthy women (Saldova *et al*., 2014) and was used to validate the results of the TIF analysis.

### 2.2 Immunohistochemistry of tissue biopsies: histological assessment and tumor subtyping

Immunohistochemistry analysis was performed as described elsewhere (Celis *et al*., 2004). First, small FFPE blocks were prepared from two to three various parts of the tissue piece and the sections were stained with a CK19 (*KRT19*) antibody. Tissue morphology, tumor cell content and visual assessment of tumor stroma percentages were evaluated as previously described (Espinoza *et al*., 2016). All slides were blindly reviewed by two independent investigators (IIG, PSG). Subtype scoring of the tumor tissues as luminal A (LumA), luminal B (LumB), luminal B HER2‐enriched (LumB HER2‐enriched), HER2, and triple negative breast cancer (TNBC) was performed based on the estrogen receptor (ER), progesterone receptor (PgR), epidermal growth factor receptor‐2 (HER2), and Ki67 status determined for each tissue sample mainly in accordance with the St. Gallen International Breast Cancer Guidelines (Esposito *et al*., 2015). The criteria used for each subtype classification are summarized in Table S1. The monoclonal mouse antibody raised against CK19 (clone 4E8) was obtained from ThermoFischer Scientific. The monoclonal mouse antibody raised against Ki67 (clone MIB‐1) was purchased from DAKO. The monoclonal antibody raised against ER (clone 1D5) was obtained from DAKO. The monoclonal antibody raised against synthetic peptide directed toward the N‐terminal end of PgR was purchased from DAKO. The polyclonal rabbit antibody raised against Her2 (Hercep Test) was obtained from DAKO. For all staining, positive control slides were included in parallel in accordance with the manufactory instructions. For the negative controls, the slides were incubated with PBS instead of primary antibodies. All information about patients and samples analyzed in the study is presented in Table S2.

### 2.3 Estimation of tumor infiltrating lymphocytes and their subpopulations

Immunohistochemistry analyses were performed to examine the most prominent components of the immune microenvironment in the corresponding tumor biopsies used for interstitial fluid recovery and molecular characterization. Scoring of total leukocytes, T lymphocytes, T helper lymphocytes, cytotoxic T lymphocytes, and macrophages were determined based on staining performed with antibodies raised against CD45+, CD3+, CD4+, CD8+, and CD68+, respectively. The monoclonal antibodies raised against CD45 (clone 2B11 + PD7/26), CD4 (clone IS 649), CD8 (clone 144B), and CD68 (clone PG‐M1) were purchased from DAKO. The polyclonal antibody raised against synthetic peptide from the intracellular part of the ε‐chain of human CD3 was obtained from DAKO. The proportion of TILs in tissue sections was evaluated in accordance with the recommendations of the International TILs Working Group 2014 (Salgado *et al*., 2015). An assessment of overall inflammatory reactions and the number of lymphoid cells present within biopsies were determined by hematoxylin and eosin (HE) staining as described elsewhere (Denkert *et al*., 2010): 1+ (>10%), 2+ (10–50%), 3+ (>50%). These scores were independently and blindly assigned two independent investigators (IIG and PSG). The macrophage marker, CD68, was also evaluated with the same criteria. For each immune cell population that was analyzed, the expression results were dichotomized as low (<10%) and high (>10%). Table S2 (columns T‐X) contains the detailed information regarding stratification of the samples based on the TILs presence.

### 2.4 Interstitial fluid recovery

Tumor interstitial fluids and NIF samples were extracted from fresh breast tumor and normal tissue specimen, as previously described (Celis *et al*., 2004). Briefly, 0.1–0.3 g of clean tissue was cut into small pieces (~1 mm^3^ each), washed twice in cold PBS to remove blood and cell debris, and then incubated in PBS for 1 h at 37 °C in a humidified CO_2_ incubator. The samples were then centrifuged at 200 ***g*** and 4000 ***g*** for 2 min and 20 min, respectively, at 4 °C. The supernatants were carefully aspirated, and total protein concentration for each sample was determined with the Bradford assay (Bradford, 1976).

### 2.5 *N‐*glycan profiling by UPLC

#### 2.5.1 Sample processing for UPLC

About 50–100 μL of TIF, NIF, depending on the original protein concentration, was lyophilized followed by resuspension in 10 μL of distilled water. *N*‐glycans were released using an updated version (Saldova *et al*., 2015) of the high‐throughput automated method described by Stockmann and coauthors (Stockmann *et al*., 2015) using a liquid‐handling robot. The samples were then denaturated with dithiothreitol, alkylated with iodoacetamide, and *N*‐glycans were released from the protein backbone enzymatically via PNGase F (Prozyme Glyco *N*‐Glycanase, code GKE‐5006D, 10 μL per well, 0.5 mU in 1 m ammonium bicarbonate, pH 8.0). The released glycans were captured on solid supports, and excess reagents, salts, and other impurities were removed by vacuum or centrifuge filtration, and the glycans were then released and labeled with the fluorophore 2‐aminobenzamide (2‐AB). Next, glycans were purified in a 96‐well chemically inert filter plate (Millipore Solvinert, hydrophobic polytetrafluoroethylene membrane, 0.45 μm pore size) using HyperSepDiol SPE cartridges (ThermoScientific, Waltham, MA, USA) (Stockmann *et al*., 2015), with each well containing all glycans released from individual sample. The samples were then lyophilized and dissolved in 10 μL of an acetonitrile–water mixture (70 : 30).

#### 2.5.2 UPLC analysis

Purified *N*‐glycans were automatically injected into the UPLS system in a mixture of 70% acetonitrile in water (see above). For UPLC analysis, a 2.1 × 150 mm HILIC column (Waters, Milford, MA, USA) was coupled with an Acquity UPLC system (Waters) equipped with a Waters temperature control module and a Waters Acquity fluorescence detector. The column temperature was set to 40 °C, and two buffer solutions, A (50 mm formic acid adjusted to pH 4.4 with ammonia solution) and B (pure acetonitrile), were used to run the following 30 min linear gradient: 0.56 mL·min^−1^ flow rate for 23 min with 30–47% of buffer A followed by 47–70% of A and finally reverting back to 30% of A to complete the run. The elution of *N‐*glycan was measured by fluorescence detection at 420 nm with excitation at 330 nm. The system was calibrated using an external standard of hydrolyzed and 2AB‐labeled glucose oligomers to create a dextran ladder, as described previously (Stockmann *et al*., 2015). The use of an external standard enabled reproducible relative quantitation of glycans between the runs. The GU (glycose unit) assigned to every peak in the chromatogram, based on the standard and each peak (collection of glycans at the same GU), is proportional of the entire glycome calculated as 100% of fluorescence intensity.

A total of 165 *N*‐glycans assigned to 46 glycan peaks (GP1 to GP46) were detected in tissue interstitial fluids. Each glycan peak (GP) contains several predominant structures. The total composition of all structures and predominant glycan features are summarized in Table S3.

#### 2.5.3 Feature analysis

*N*‐glycan peaks (GP) were pooled based on similar structural or compositional features of the peak glycan members. Features relating to a peak were determined based on the major glycan members of that peak described at Saldova *et al*. (2014).

### 2.6 Bioinformatic and statistical analysis

#### 2.6.1 Curation of the dataset for analysis

The analysis of glycan abundances was performed using two datasets in parallel; (d1) all available samples corresponding to 85 TIF samples and 54 NIF samples and (d2) paired tumor and normal samples including a total of 54 individual TIF–NIF pairs (108 samples). The peaks of the glycan UPLC output represent the relative area for each glycan peak in the spectrum. The glycan abundances were log2‐transformed to reduce the impact of outliers and to deal with the skewness of the glycan distribution. The log2 transformation resulted in the majority of glycan abundances approaching normal a distribution. After log2 transformation, the data were corrected for batch effects using the ComBat function of the sva r package (Johnson *et al*., 2007). ComBat batch‐corrected data were only used for plotting purposes.

All the initial data, scripts for analyses, and outputs are released as free materials at https://github.com/ELELAB/N-glycan-TIF so that our findings could be reproduced.

#### 2.6.2 Multidimensional scaling

Classical multidimensional scaling (r version 3.3.1) was used to reduce the number of the dimensions within the data. Specifically, 108 samples (54 TIF–NIF pairs) with each 63 measurements of glycan/glycan feature abundances were reduced to two dimensions (M1 and M2). Multidimensional scaling (MDS) was performed with the function cmdscale() using Euclidean distance as the distance metric. The plotting was done with r package ggplot2 2.2.1.

#### 2.6.3 Differential abundance analysis

The differential abundance analysis (DAA) was performed using the statistical software limma (Linear Models for Microarray Data) implemented in r (Ritchie *et al*., 2015). limma has few underlying statistical assumptions and is known to be powerful for small sample sizes as a result of shrinkage of feature‐specific variances (Ritchie *et al*., 2015). Although limma was originally developed for analysis of microarray, a number studies had shown the versatility of this software for the analysis of other ‐omics data (Castello *et al*., 2012).

For the analysis of paired data (d2), the information on patient ID was incorporated into the design matrix to account for patient‐specific effects. For the analysis with unpaired data (d1), information on batch was added to the model. We carried out DAA between NIF samples and TIF samples using a corrected *P*‐value (FDR: false discovery rate) of 0.05 as the cutoff for significance.

To determine whether any clinical variables could be related to the abundance of specific *N*‐glycans groups or *N*‐glycan features, DAA was performed for tumor grade (Gr), receptor status (HER2, ER, PgR), and tumor infiltrating lymphocyte status (TILs, CD3, CD4, CD8, CD45, CD68) in the sample (see Table S2).

All analyses were performed using two datasets in parallel; (d1) all available samples corresponding to 85 TIFs (stratifying into luminal A = 43, luminal B = 12, luminal B HER2‐enriched = 8, HER2 = 9, and TNBC = 13) and 54 NIF samples, and (d2) paired tumor and normal samples including a total of 54 individual TIF–NIF pairs (108 samples): luminal A = 22, luminal B = 10, luminal B HER2‐enriched = 6, HER2 = 4, and TNBC = 12 (see Table S2).

#### 2.6.4 Hierarchical clustering analysis

Hierarchical clustering was performed to visually inspect the results of the DAA. Agglomerative hierarchical clustering was implemented in R with hclust() (R‐stats), using the Ward's method (ward.2D), the statistical premise of which is to minimize the total within‐cluster variance (Murtagh and Legendre, 2014).

#### 2.6.5 Correlation analysis—glycan abundances in TIF and serum

The log2‐transformed abundances of TIF *N*‐glycans structures were correlated with corresponding serum profiles of 28 serum samples available. Classic Pearson's product–moment correlation was performed in r. The significance of correlation scores was tested and obtained *P*‐values corrected using FDR. Correlations with an FDR < 0.05 were considered significant and kept for further analysis.

#### 2.6.6 Survival analysis

Survival analysis was performed using a Cox proportional hazard model. Dataset d1 (e.g., all available TIF, *n* = 85) was used for analysis. Survival was modeled using one *N*‐glycan feature at a time, for example, not accounting for potential inter‐glycan abundance effects. Clinical parameters where tested for confounding effects on *N*‐glycan levels and/or clinical outcome. As expected, age at diagnosis was found to have a significant effect on overall survival. Of the remaining parameters, TIL status (CD4^+^ and CD45^+^) was found to be a confounder. Before regression analysis, the covariates were tested for violation of the proportional hazard assumption. Also, the log‐linearity of continuous variables (*N*‐glycans and age) was evaluated. In the final models, age was modeled with splines (df = 2). Four confounder packages were tested accounting for an age at surgery and/or tumor infiltrating lymphocytes status (total TILs, CD4^+^, and CD45^+^)—GPX represents a glycan peak:

cpk1=GPX+Age

cpk2=GPX+Age+TILs

cpk1=GPX+Age+CD4

cpk2=GPX+Age+CD45

In addition to the cox regression model with overall patient survival as outcome (censored = 0 and event = 1), survival analysis was performed using, as events, only those deaths for which information on primary cause of death was available and denoted as ‘malignant neoplasm of breast’ (censored = 0 and malignant neoplasm of breast = 1).

Results of the cox models were reported as hazard ratios, confidence intervals, and FDR values. Survival curves were generated using the corrected regression models. Survival curves were made assuming an age of 66 at surgery (median age at entry for the cohort). For each *N*‐glycan composition, high abundance was defined as the upper 25th percentile, while low abundance was defined as the lower 25th percentile. Survival analysis was performed using r‐packages survcomp and survminer (Haibe‐Kains *et al*., 2008).

**Figure 1:**
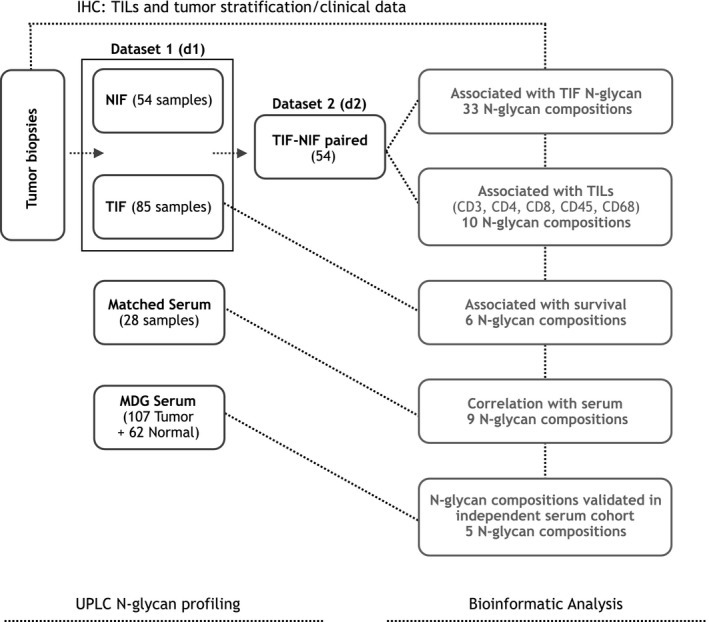
The experimental workflow including final number of samples used in each analysis.

The experimental workflow including number of samples used in each analysis is presented in Fig. 1.

## 3 Results

### 3.1 Comparative analysis of *N*‐glycan structures in matched TIF and NIF: distribution across five BC subtypes and correlation with clinicopathological parameters

**Figure 2:**
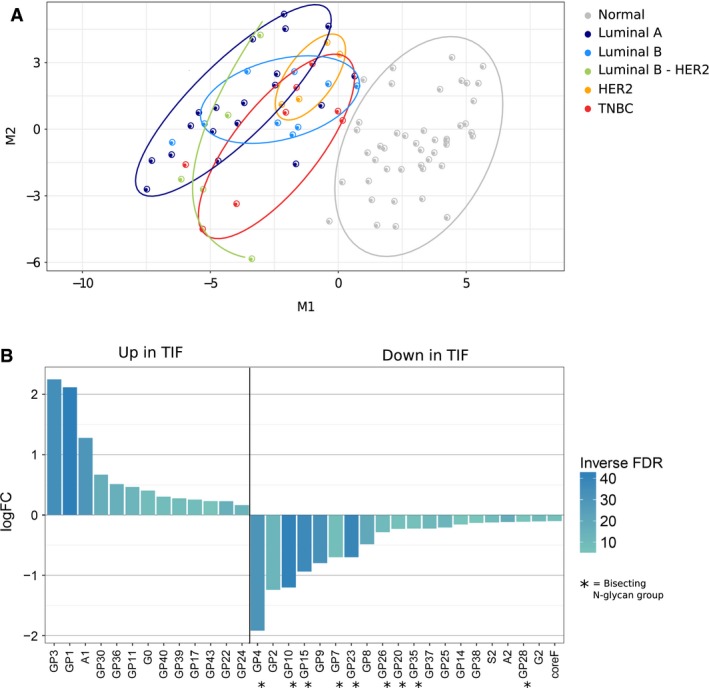
*N*‐glycans with differential abundance between TIF and NIF. (A) Multidimensional scaling plot. M1 and M2 are the scaling components that best captured the distance (squared euclidian) relationship between samples. Gray points denote NIF samples; colored points are TIFs, stratifying into BC subtypes luminal A (LumA), luminal B (LumB), luminal B HER2‐enriched (LumB HER2‐enriched), HER2, and TNBC. Ellipses capture the majority of samples within a single group. (B) Bar plot shows 33 *N*‐glycan groups with differential abundance between TIF and NIF. Height and directionality of bars indicate the log‐fold change; color depicts the scaled inverse FDR: Darker shade indicates a lower FDR. All glycans depicted in the plot had FDR ≤ 0.05. Stars mark peaks containing bisecting *N*‐glycans.

To obtain a general overview of *N*‐glycan profiles across TIF‐ and NIF‐matched counterparts, we plotted all paired samples using multidimensional scaling (MDS). Forty‐six glycan groups (GP1–GP46) and 17 *N*‐glycan features were quantified (Table S3). The MDS plotting revealed considerable segregation of TIF and NIF samples (Fig. 2A).

To evaluate a possible segregation of glycan patterns across five main BC subtypes, we stratified tumor samples in accordance with the St. Galen criteria: luminal A, luminal B, luminal B HER2‐enriched, HER2, and TNBC (Esposito *et al*., 2015). As seen in Fig. 2A, no clear clustering between subtypes was identified (Fig. 2A), even after merging samples into three major groups: luminal, HER2, and TNBC (data not shown). The absence of a significant difference in *N*‐glycan abundance across subtypes may be partly explained by a large difference in the numbers of samples in each subtype group (Table S2). We speculate that the partitioning of BC samples into subtypes based on immunohistochemistry is not directly transferrable to *N*‐glycan abundance and/or that *N*‐glycan levels may reflect an alternative glycan‐based tumor stratification.

We also did not find any significant correlation between the abundance of TIF *N*‐glycan structures and clinical tumor variables including grade, type, and/or hormone or growth receptor status (data not shown). These results indicate that *N*‐glycans externalized into breast tumor interstitial fluid may not be directly associated with these clinicopathological characteristics of the tumor.

In order to determine which *N*‐glycans were differentially represented in fluids originating from tumor compared to normal breast tissue, we performed differential abundance analysis (DAA) using paired TIF–NIF samples. In accordance with the clustering observed in the MDS plot (Fig. 2A), DAA yielded 33 *N*‐glycan groups with significantly differential abundance in TIF vs. NIF: 13 groups with significantly elevated levels in TIF samples and 20 groups with significantly decreased levels in TIF as compared to NIF counterparts (Fig. 2B, Table 1). Our results showed that TIF samples were enriched for particular type of sialylated (S3–S4), highly galactosylated (G3–G4) *N*‐glycans with a high number of antennae (A3–A4), as well as for simpler *N*‐glycans (such as monoantennary glycan, A1, no galactosylation, G0). A significant decrease in core fucosylated and bisected *N*‐glycans, represented by GP4, GP7, GP10, GP15, GP20, GP23, GP26, GP28, and GP35, was observed in TIF. DAA with all available samples (d1 set—see Material and methods 6.1) yielded a set of *N*‐glycans almost identical to the one obtained with paired samples only (data not shown).

**Table 1:** *N*‐glycans with differential abundance in TIF and NIF. Statistics for each differentially abundant *N*‐glycan group and feature (log‐fold change, *P*‐value, and FDR), as well as directionality in TIF (up or down). Bisected *N*‐glycans are highlighted in bold

Glycan label	logFC	*P‐*Value	FDR	Direction in TIF samples	Annotation of major *N*‐glycan structures in corresponding GP	Structures
GP3	2.25	3.3e‐12	4.2e‐11	Up	A1[6]G1	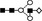
GP1	2.11	1.9e‐15	1.2e‐13	Up	A1	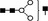
A1	1.28	1.2e‐10	9.5e‐10	Up		
GP30	0.67	1.6e‐06	9.4e‐06	Up	A4G4S[3]1	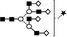
GP36	0.51	3.8e‐06	1.8e‐05	Up	FA3G3S[3,6,6]3	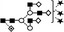
GP11	0.47	3.8e‐05	1.4e‐04	Up	M6 D1 D2	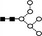
G0	0.41	6.8e‐04	2.0e‐03	Up		
GP40	0.30	7.7e‐04	2.1e‐03	Up	A4F1G3S[3,3,3]3 A4F1G3S[3,3,6]3 A4F1G3S[3,6,6]3	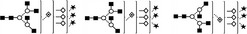
GP39	0.27	8.6e‐04	2.3e‐03	Up	A4G4S[3,3,6]3; A4G4S[3,6,6]3	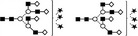
GP17	0.26	9.2e‐04	2.3e‐03	Up	FA2[3]G1S[6]1	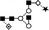
GP43	0.23	1.4e‐02	2.9e‐02	Up	A4G4S[3,3,3,6]4	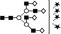
GP22	0.23	3.6e‐06	1.8e‐05	Up	FA2G2S[6]1	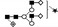
GP24	0.17	1.4e‐05	6.5e‐05	Up	A2G2S[3,6]2	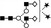
**GP4**	−1.92	8.1e‐11	7.3e‐10	Down	A2B	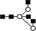
GP2	−1.24	3.8e‐05	1.4e‐04	Down	FA1	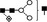
**GP10**	−1.20	1.0e‐14	3.2e‐13	Down	FA2[6]BG1	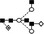
**GP15**	−0.94	4.2e‐13	6.6e‐12	Down	FA2BG2	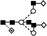
GP9	−0.79	8.2e‐12	8.6e‐11	Down	FA2[3]G1	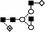
**GP7**	−0.70	1.4e‐03	3.5e‐03	Down	A2[6]BG1	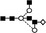
**GP23**	−0.70	8.5e‐14	1.8e‐12	Down	FA2BG2S[3]1	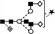
GP8	−0.49	1.3e‐07	9.3e‐07	Down	FA2[6]G1	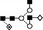
**GP26**	−0.28	3.3e‐04	1.1e‐03	Down	A2BG2S[3,6]2	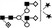
**GP20**	−0.23	4.1e‐03	9.0e‐03	Down	A2BG2S[6]1	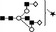
**GP35**	−0.23	3.5e‐03	7.9e‐03	Down	A3BG3S[3,3,3]3	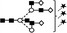
GP37	−0.23	1.0e‐04	3.5e‐04	Down	A3F1G3S[3,3,3]3	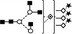
GP25	−0.21	3.9e‐05	1.4e‐04	Down	A2G2S[3,6]2	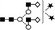
GP14	−0.16	1.6e‐02	3.1e‐02	Down	FA2G2	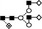
GP38	−0.13	2.5e‐02	4.8e‐02	Down	A4G4S[3,3,3]3	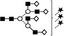
S2	−0.12	5.3e‐04	1.6e‐03	Down		
A2	−0.12	7.2e‐07	4.6e‐06	Down		
**GP28**	−0.11	1.5e‐02	3.0e‐02	Down	FA2BG2S[6,6]2	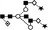
G2	−0.11	3.1e‐04	1.0e‐03	Down		
coreF	−0.10	2.2e‐03	5.1e‐03	Down		

### 3.2 Association of *N*‐glycan pattern with TILs

Tumor infiltrating lymphocytes have been shown to play an essential role in BC progression, influencing cross talk between tumor and stromal cells and providing prognostic and potentially predictive values (Bedognetti *et al*., 2016; Ingold Heppner *et al*., 2016). To disclose a possible relationship between *N*‐glycan structures, which displayed differential abundance between TIF and NIF (Fig. 2B) with the composition of TILs within tumor microenvironment (Table S2 for details), we performed DAA, considering the extent of lymphocyte infiltration within tumor biopsies. A detailed evaluation of TIL subtypes, often described in breast cancer literature, was performed by IHC using the antibodies specific for particular lymphocyte antigen (Fig. S1).

**Figure 3:**
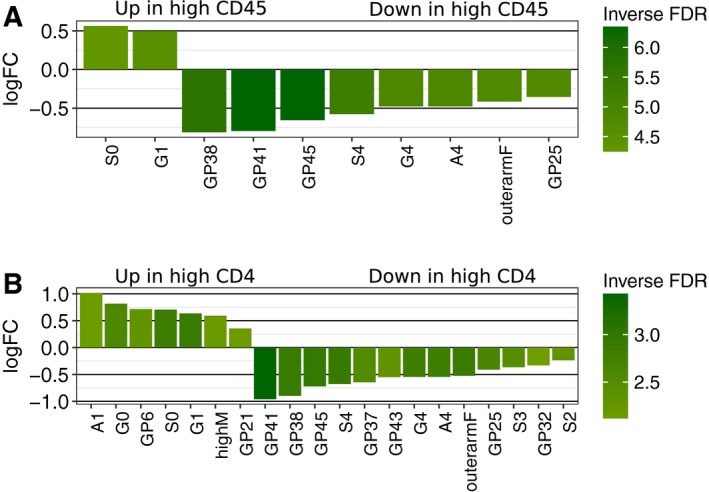
*N*‐glycans with differential abundance in TIL‐enriched and TIL‐depleted samples. (A) Bar plot shows *N*‐glycan groups with differential abundance in tumor samples with low (0/+1) and high (+2/+3) overall TIL status, as determined by CD45 positivity (see Table S2 for details). (B) Bar plot shows *N*‐glycan groups with differential abundance in samples with low vs. high TIL status, as determined by CD4 positivity. Height and directionality of bars indicate log‐fold change. Shade depicts inverse FDR: Darker shade indicates lower FDR. All *N*‐glycans depicted in the plot had FDR ≤ 0.05.

The association between *N*‐glycans and overall TILs determined by CD45^+^ revealed ten *N*‐glycan groups, which were differentially abundant in TIF released from biopsies enriched for TILs compared to samples with low TIL status (Fig. 3A). In particular, it turned out that samples with high levels of lymphocyte infiltration (see Table S2 for details) showed significantly high levels of G1 (glycans with 1 galactose) and S0 (glycans without sialic acid), whereas GP38 (mostly tetraantennary tetragalactosylated trisialylated glycans, A4G4S3), GP41 (mostly tetraantennary tetragalactosylated tetrasialylated glycans), GP45 (mostly outerarm fucosylated tetraantennary tetragalactosylated tetrasialylated glycans), S4 (tetrasialylated glycans), G4 (tetragalactosylated glycans), A4 (tetraantennary glycans), GP25 (mainly biantennary digalactosylated disialylated glycans without fucosylation, A2G2S2), and outerarmF (outerarm fucosylated glycans) were predominant in samples with low level of lymphocyte infiltration (Fig. 3A and Table S4).

To determine which subpopulation of TILs contributed most to the differential abundance of *N*‐glycoforms in tumor proximal fluid, glycome profiles were analyzed relative to four TIL subgroups: CD3^+^, CD4^+^, CD8^+^, and CD68^+^. The results showed that total (CD45 + ) and T helper cells (CD4^+^)‐enriched biopsies displayed relatively similar *N*‐glycan patterns (Fig. 3A,B). No significant differential abundance was observed for CD3+, CD8+, and CD68+ TIL subpopulations (data not shown). A high abundance of A1 (monoantennary glycans), G0 (agalactosylated glycans), GP6 (mainly high‐mannose glycans, M5), highM (total high‐mannose glycans), and GP21 (mainly biantennary monosialylated glycans [A2G2S1] and high mannosylated hybrid glycans [M5A1G1S[3]1]) as well as S0 and G1 was associated with CD4^+^‐enriched samples. Inversely, samples with high CD4^+^ had lower levels of multibranched *N‐*glycans and highly sialylated *N‐*glycans, many of which contain outerarm fucose (e.g., GP25, GP32, GP37, GP38, GP41, GP43, GP45) peaks as well as S4, G4, A4, outerarmF, S3, and S2 features.

### 3.3 Prognostic potential of TIF N‐glycan abundances

To determine whether *N*‐glycan abundance predicted outcomes for patients with BC, overall survival analysis was performed across all patients for which survival information was available with a Cox proportional hazard model. Two types of Cox models were compared: one corrected only for age at diagnosis and one corrected for age at diagnosis + TIL status (overall, estimated by CD45^+^ and CD4^+^).

**Figure 4:**
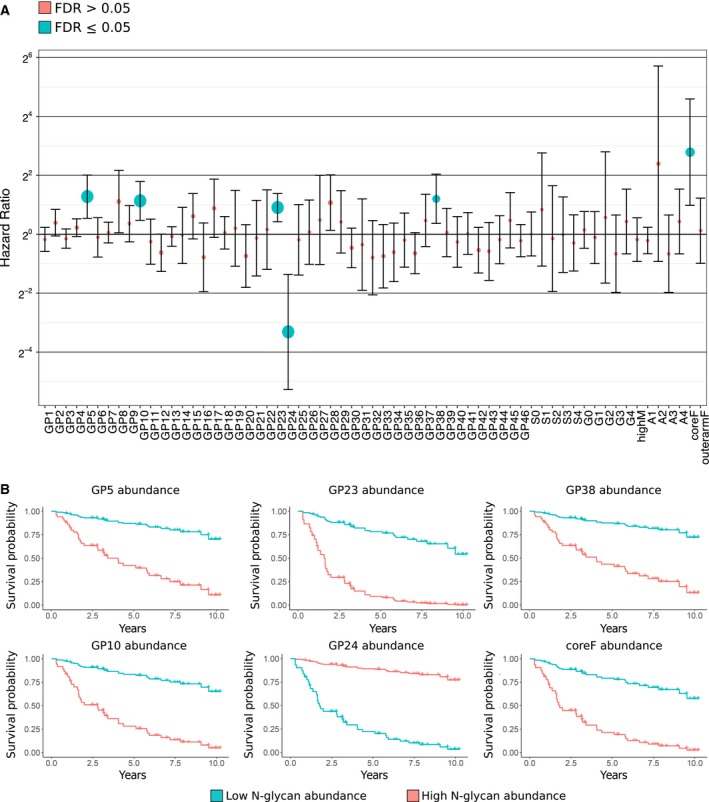
Prognostic association of TIF *N*‐glycans with overall survival. (A) Cox proportional hazards regression. Dot plot shows hazard ratios and confidence intervals for each *N*‐glycan group. Dot size indicates the inverse FDR, that is, small FDRs are depicted as large dots. Dot color indicates whether the abundance of a given *N*‐glycan was significantly associated with overall survival (FDR ≤ 0.05). (B) Survival curves based on low and high abundance of five *N*‐glycan groups associated with poorer overall survival and one *N*‐glycan group (GP24) found to be protective. Curves are fitted (simulated) using the age at diagnosis of 66 years (median of observed data). Curves show the probability of survival in years after diagnosis (at the age of 66) as a function of a high (orange) or low (blue) level of a given *N*‐glycan (minimum observed value—lower 25th percentile, and maximum observed value—upper 25th percentile for the *N*‐glycan in question).

The Cox model, corrected only for age at diagnosis, yielded six *N*‐glycan groups, which were significantly associated with overall outcome (Fig. 4). One of these, GP24 (biantennary bigalactosylated bi‐sialic‐acid glycan, A2G2S2), had a hazard ratio below 1, for example, a high level of GP24 was predictive of superior prognosis. The remaining five groups had hazard ratios greater than 1, implying that a high abundance of these was associated with poor outcomes. These included GP5 (core fucosylated biantennary glycan, FA2), GP10 (core fucosylated bisected biantennary monogalactosylated glycan, FA2[6]BG1), GP23 (core fucosylated bisected biantennary bigalactosylated monosialylated glycans, FA2BG2S1), GP38 (mostly tetraantennary tetragalactosylated trisialylated glycans, A4G4S3), and coreF (core fucosylated glycans) (Fig. 4A). All glycans, except GP5, were among those that segregated TIF and NIF.

The results of the survival analysis, in which a death was only classified as an events if the cause of death was known to be ‘malignant neoplasm of breast’, yielded a similar pattern as the one observed for the cox model with overall survival, with *N*‐glycans GP5, GP8, GP10, GP23, GP38, and coreF displaying high hazard ratios (HR: ~ 2.0 ‐ 7.0) (Fig. S2). However, despite the high HRs and the fact that the 95% confidence intervals of these *N*‐glycans did not overlap 1, *P*‐values were no longer significant after correction for multiple testing (FDR). We attribute this lack of significance to the lower power associated with this model, for example, if only outcomes with known cause of death are classified as events, the ratio of events/censures is notably reduced—this will have a large impact on a small(er) dataset.

Correction for TILs estimated by CD45^+^ or CD4^+^ did not alter the overall results of survival analysis. Figure 4B shows the survival curves using the age‐corrected regression model for each structure associated with overall survival. The association of low GP10 and GP23 levels with poorer outcome is in agreement with the functional role of bisecting *N*‐glycans in tumor development.

### 3.4 Correlation of N‐glycan abundance in paired TIF and serum

To identify *N*‐glycans with potential as noninvasive blood‐based biomarkers for breast malignancy, we determined which of the 33 *N‐*glycans displayed a significant correlation of abundances within 28 paired serum samples, after segregating NIF and TIF samples (Table 1, Fig. 2B).

**Figure 5:**
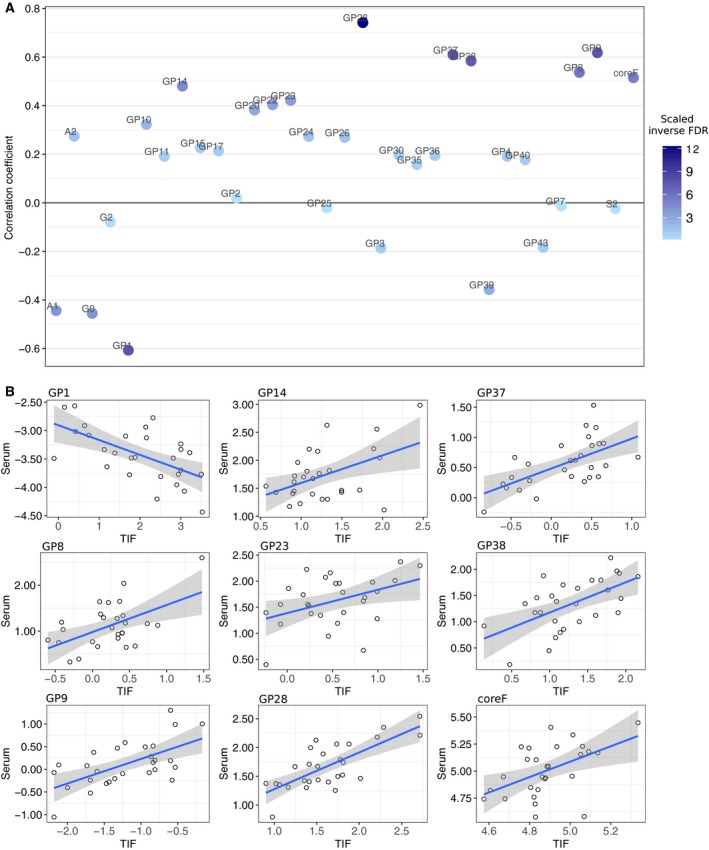
Correlation of *N*‐glycan abundance in TIF and matched serum samples. (A) Dot plot shows Pearson's correlation coefficient between *N*‐glycan abundances in serum and TIF. Shade of dot represents inverse FDR. (B) Scatter plots of nine *N*‐glycan groups with abundances significantly correlated in serum and TIFs. Line color indicates directionality (red = negative correlation, blue = positive correlation). Gray shading shows the confidence of the regression line in a given area of the plot.

Classic Pearson's product–moment analysis was performed to correlate *N*‐glycan structures in TIF and serum. Of 33 *N*‐glycan groups, nine were correlated with *N*‐glycan levels in serum (Fig. 5, Table S5).

To determine whether any of the *N*‐glycans present in serum reflect tumor immune status, we performed correlation analysis for the overall proportion of TILs (CD45+) and T helper cells (CD4+) within corresponding tumors. Two *N*‐glycan groups, GP37 (mostly triantennary outerarm fucosylated trigalactosylated trisialylated glycans, A3F1G3S3) and GP38 (mostly tetraantennary tetragalactosylated trisialylated glycans, A4G4S3), were less abundant in serum from patients exhibiting high overall levels of TIL (CD45^+^) or T helper cells, as determined by CD4^+^ staining in corresponding tumors (Fig. 3, Table S5). *N*‐glycan GP23, GP38, and coreF structures were identified as predictive for overall survival (Fig. 4), demonstrating a significant correlation of abundance between TIF and paired serum (see Table S5 for details).

### 3.5 Validation of *N*‐glycan structures in an independent serum cohort

To validate the results obtained from glycan profiling of matched TIF/serum samples, 33 DA *N*‐glycans were analyzed across an independent serum MDG dataset. The MDG cohort contains samples obtained from healthy controls and BC patients (Saldova *et al*., 2014) and profiled with the same UPLC‐based technology, thus minimizing technical variability between experimental platforms. DAA was applied to the MDG dataset on log2‐transformed data, in agreement with the protocol applied to our TIF–NIF BC cohort.

Twelve of 33 glycan groups were found to be abundant in both TIF and MDG BC serum samples as compared to NIF and MDG normal serum (Table 2). Specifically, we found *N*‐glycan peaks for GP3 (monoantennary monogalactosylated glycan, A1[6]G1), GP30 (tetraantennary tetragalactosylated monosialylated glycan, A4G4S[3]1), GP39 (tetraantennary tetragalactosylated trisialylated glycans, A4G4S3), and GP40 (mostly tetraantennary outerarm fucosylated trigalactosylated trisialylated glycans, A4F1G3S3) to be more abundant in TIF and BC serum samples as compared to NIF and normal serum samples. Inversely, bisected biantennary core fucosylated *N*‐glycans (GP8, GP9, GP10, GP14, GP15, G23, as well as A2 and fucosylated glycans [coreF]) were less abundant in TIF and BC serum compared to normal samples.

**Table 2:** Differential abundance of *N*‐glycan groups segregating TIF, NIF, matched serum, and MDG cancer and normal serum. Column 3 = *N*‐glycan rank in TIF based on log‐fold change, in total: 13′*N*‐glycan groups increased in TIF; 20 *N*‐glycan groups decreased in TIF (see Table 1). Column 4 = *N*‐glycan rank in MDG cancer serum based on log‐fold change, in total: 26 *N*‐glycan groups increased and 18 *N*‐glycan groups decreased. Column 5 indicates correlations between abundance of *N*‐glycan composition in TIF and paired serum samples. DA, differential abundance. *N*‐glycan groups for which abundance in MDG correlated with abundance in TIF‐paired serum datasets are highlighted in bold

Glycan label	Direction in TIF and MDG cancer serum	Rank in TIF out of total number of DA *N*‐glycans	Rank in MDG cancer serum out of total number of DA *N*‐glycans	Significant correlation between TIF and matched serum
GP3	Up	1|13	12|26	No
GP30	Up	4|13	24|26	No
GP40	Up	8|13	15|26	No
GP39	Up	9|13	7|26	No
GP10	Down	3|20	14|18	No
GP15	Down	4|20	9|18	No
**GP9**	Down	5|20	6|18	Yes
**GP23**	Down	7|20	3|18	Yes
**GP8**	Down	8|20	4|18	Yes
**GP14**	Down	14|20	1|18	Yes
A2	Down	17|20	18|18	No
**coreF**	Down	20|20	5|18	Yes

Five of 12 *N*‐glycan groups (highlighted in bold in Table 2) displayed a significant correlation of abundance in MDG‐ and TIF‐paired serum datasets. Segregation analysis based on these groups (GP8, GP9, GP14, GP23, and coreF) revealed a significant separation between normal and cancer MDG serum samples and showed a significant correlation in matched serum (Fig. S3).

## 4 Discussion

**Figure 6:**
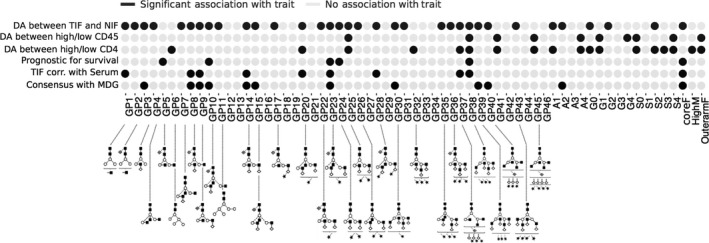
Overview of the main results obtained in the study. *N*‐glycan groups (1–46) and *N*‐glycan features. Rows = traits and columns = *N*‐glycans ID. Black dots denote which *N*‐glycan group and features were significantly associated with a given trait based on analysis described in the corresponding result sections. The prominent *N*‐glycan structures within a given *N*‐glycan peak are specified below the GP's ID.

To the best of our knowledge, this study is the first analysis of the *N*‐glycome in the tumor interstitium of patients with BC. Experiments were designed to identify aberrant glycosylation associated with tumor growth and progression. The study is part of a comprehensive project focused on characterization of the entire molecular complement of breast tumor interstitial fluid, aiming to identify integrated signatures associated with events underlying breast tumor metabolism, as detectable in blood (Espinoza *et al*., 2016; Halvorsen *et al*., 2017). The analysis included a detailed morphological characterization of tumor lesions and evaluation of the spatial heterogeneity of TILs in tumor specimens, to elucidate the influence of tumor immune composition on secreted *N*‐glycome complement. Data were subjected to bioinformatics analysis to characterize the *N*‐glycome in the breast tissue interstitium and to reveal potentially valuable correlations of aberrant glycan patterns with breast tumor biology, including clinical outcome and presence in the blood. Finally, data were computationally validated with the independent serum MDG dataset (Saldova *et al*., 2014), which contains BC carcinoma and nonmalignant serum profiled by analogous technology. Figure 6 summarizes the main results of our analyses.

### 4.1 *N*‐glycan patterns in TIF

Multidimensional scaling and DAA of *N*‐glycan profiles revealed distinct segregation between TIF and NIF. We detected 33 *N*‐glycan groups and features with differential level between groups. *N*‐glycan structures displaying significantly higher abundance in TIF as compared to NIF belong mainly to the monoantennary type [GP1, GP3, and A1 (sum of all monoantennary glycans)]. Our results are consistent with those of a recent study reporting a clear segregation of *N*‐glycans circulating in the blood of patients with BC and normal individuals (Saldova *et al*., 2014), particularly, for the core fucosylated *N*‐glycans (Hamfjord *et al*., 2015; Kizuka and Taniguchi, 2016) that are represented by a set of GP4, GP7, GP10, GP15, GP20, GP23, GP26, GP28, and GP35 structures in our TIF samples.

It has previously been shown that specific glycan structures have different impacts on cell adhesion, which is one of the major molecular events during malignant transformation that affects cancer cell fate (Moremen *et al*., 2012). Interestingly, we found nine bisecting structures (GP4, GP7, GP10, GP15, GP20, GP23, GP26, GP28, and GP35) to be significantly more abundant in NIF. The observed depletion of bisecting *N*‐glycans in TIF is in agreement with current consensus regarding the functionality and role of *N*‐bisecting glycans in cancer progression. Recent research has shown that extension of GlcNAc bisecting has a significant effect on cell survival and tumor aggressiveness (Kizuka and Taniguchi, 2016). This phenomenon has also been reported for cadherins, proteins that have a substantial impact on cell adhesion. When modified by bisecting glycans, cadherins reinforce cell adhesion and are consequently associated with cancer suppression. In contrast, cadherins bearing branched complex *N*‐glycans are less involved in the control of cell adhesion and are associated with cancer progression (Carvalho *et al*., 2016). It has been proposed, mainly by using *in vitro* model systems, that the presence of this unique bisecting structural feature has important implications for the entire cellular glycan complement. Thus, enzymes responsible for producing *N*‐glycan groups other than those with bisecting branches (e.g., GnT‐IV, GnT‐V) are almost completely inhibited by the presence of a bisecting GlcNAc residue in the *N*‐glycan molecule (Stanley *et al*., 2009). The results of our study support this notion, clearly demonstrating a significant abundance of particular set of bisecting glycan species in the interstitium of nonmalignant lesions as compared to their neoplastic counterparts.

### 4.2 Correlation between TILs and *N*‐glycan composition

Tumor infiltrating lymphocytes are frequently found within tumors, suggesting that tumors trigger an immune response in the host. The presence of TILs within the tumor microenvironment has been reported as an important biomarker linked to clinical outcome (Ingold Heppner *et al*., 2016). In this study, we immune‐profiled particular subsets of TILs, for example, those most often described in connection with BC in current literature (Denkert *et al*., 2010; Salgado *et al*., 2015).

We identified a number of glycoconjugates in TIF that were significantly associated with the proportion of TILs (Fig. 3), as determined by immunohistochemistry. In samples with high levels of total TILs, we observed an increase in simple high mannose *N*‐glycan features (G0, G1, S0, and highM) and, inversely, a decrease in the abundance of highly complex *N*‐glycan groups (A4, G4, S4, GP25, GP37, GP38, GP41, GP45, etc.). To our knowledge, our data are the first evidence highlighting a direct impact of the tumor immune complement on the secreted *N*‐glycan profile in breast tumor.

### 4.3 Relationship between *N*‐glycan patterns and clinical outcome

Our survival analysis with overall patient survival as outcome, showed a significant association between *N*‐glycome profiles and the overall survival of patients with BC. Cox proportional hazard regression revealed five *N*‐glycan peaks to be significantly associated with poor survival (GP5, GP10, GP23, GP38, and coreF) and one glycan peak (GP24) as predictive of positive clinical outcome (Fig. 4). All glycan peaks, except GP5, were among those that segregated TIF and NIF. We speculate that the absence of GP5 (core fucosylated biantennary glycan, FA2) among the 33 differentially abundant glycans segregating TIF and NIF may be related to the fact that only a subset of patients exhibit high abundance of this *N*‐glycan, which is prognostic for overall survival according to our analyses. Indeed, the remaining patients display GP5 levels similar to those observed in NIF. Thus, the differential abundance of GP5 will not be detected when stratifying NIF and TIF by DAA.

The cox model in which deaths were classified as events only if cause of death was known and annotated as ‘malignant neoplasm of breast’ did not yield significant results after FDR correction (Fig. S2). Although it might be that the *N*‐glycan groups identified as prognostic from the cox model with overall patient survival have inflated *P*‐values and may be considered as partly false positives, we hypothesize that the observed lack of significance merely reflects the decrease in power (larger confidence intervals) of this model, for an already small dataset. This observation is supported by the fact that the two cox models show similar results in terms of *N*‐glycans groups identified as having the highest hazard ratios (GP5, GP10, GP23, GP38, and coreF), with significant *P*‐values before FDR correction. We cannot say for sure that patients, for which we do not have cause of death, did not die due cancer presence even if the primary cause of death is not BC itself, but a ‘side effect’ of disease. The later notion might still be of interest for prediction of patient prognosis by using identified *N*‐glycan signature.

Although correction for TIL status (CD45^+^ or CD4^+^) did not affect the overall results of survival analysis, the corrected *P*‐value for GP38 did decrease when TILs were added to the model, highlighting the relationship between GP38 levels and TIL status seen in DAA. Reduced levels of GP38 correlated with a high proportion of overall and CD4^+^ TILs, which contribute to tumor suppression (Zanetti, 2015), thus supporting an association between tumor immune status and overall survival in patients with BC. Identification of GP10 and GP23 in relation to clinical outcome supports the suggested ‘protective’ status of bisecting glycans (Kizuka and Taniguchi, 2016), whereas decreased levels of coreF have recently been reported to contribute to the malignancy of gastric cancer (Zhao *et al*., 2014).

### 4.4 Correlation of *N*‐glycan abundance in TIF and serum samples

Changes in *N*‐linked glycan structure in serum or plasma of patients diagnosed with breast, prostate, ovarian, pancreatic, liver, or lung cancer have recently been reported (Lan *et al*., 2016 and references therein). Alterations in the *N*‐glycome profile may be the result of a primary response or a general systemic reaction of the body to the progression and metabolism of a tumor. Additionally, the high degree of complexity and dynamic range of biomolecules externalized physiologically to the blood from other tissues can mask molecules released from the primary tumor. Comparative TIF serum analysis helps to discriminate biomolecules released directly from primary tumor into the tumor interstitium from the systemic body response. In this study, of 33 *N*‐glycans that segregated TIF and NIF samples, levels of nine glycan groups (GP1, GP8, GP9, GP14, GP23, GP28, GP37, GP38, and coreF) were significantly correlated with *N*‐glycan levels in serum (Fig. 5). One of these *N*‐glycan groups, GP1 (monoantennary glycan, A1), displayed an inverse significant correlation with corresponding levels in serum, that is, high levels in TIF corresponded to low levels in serum. This observation may indicate that the molecules carrying this particular glycan feature accumulate within the tumor interstitium as a primary tumor response; however, this process is not associated with subsequent transport into the bloodstream. A more detailed look at the *N*‐glycan profiles in TIF and NIF showed that two other *N*‐glycan groups, GP3 and A1, with high TIF abundance (Fig. 2B) also exhibited a negative association with corresponding serum levels, although these trends were not significant (*P* = −0.19 and −0.44, respectively). The majority of *N*‐glycans detected in these peaks are all core fucosylated biantennary except for GP38. Our findings support previous reports describing the decreased levels of some core fucosylated glycans in the sera of patients with BC (Saldova *et al*., 2014). The low levels of these types of *N*‐glycans in the sera of patients with BC may indicate that biomolecules in the tumor interstitium bearing these *N*‐glycan structures do not reach the bloodstream, but, rather, are involved in intercellular cross‐communication within the local tumor space. This assumption may be supported by the functional features reported for core fucosylated glycans (Miyoshi *et al*., 2008). Alternatively, the inverse association between particular glycans in serum and TIF may be the result of a high dilution factor as well as the expected presence of other, more abundant, glycan species originated from no tumor sites, thus masking the presence of tumor‐derived biomolecules in the blood.

Computational validation of the paired TIF–NIF serum data was achieved through comparison with the independent *N*‐glycan MDG serum dataset (Saldova *et al*., 2014). Among the nine *N*‐glycan groups, levels of which in TIF were significantly correlated with those in matched serum samples, five (GP8, GP9, GP14, GP23, and coreF) were validated within the MDG serum dataset. The fact that we did not detect more overlaps in this validation experiment may be explained by the fact that (in contrast to the MDG serum dataset) our TIF‐matched serum dataset did not include blood samples from healthy individuals, which are important when establishing the correct baseline for normality.

Levels of most biantennary glycans, such as α2,3 sialic acid‐modified *N‐*glycan chains, decreased in the sera of patients with BC in this study, that is in agreement with previously reported data. The opposite trend was observed in the sera of lung cancer patients, which is characterized by a high level of biantennary *N*‐glycan chains containing Sialyl Lewis structure (SLex) (Lan *et al*., 2016). Serum levels of biantennary *N*‐glycan chains carrying core fucose or both core fucose and sialic acid, as well as the level of complex triantennary *N*‐glycan containing only one sialic acid or both fucose and sialic acid, were decreased in tumor samples as compared to normal controls.

## 5 Conclusions

The results of our study showed (a) clear segregation of patterns of *N*‐glycan release from tumor vs. normal mammary tissue; (b) elevated levels of particular bisecting glycans (GP4, GP7, GP10, GP15, GP20, GP23, GP26, GP37, and GP28), which contribute to tumor suppression in normal breast tissue interstitium; (c) association of several *N*‐glycans (A1, G0, GP6, M5, highM, GP21, GP41, GP38, GP45, GP37, GP43, GP26, GP32, and S2) in breast tumor interstitium with the proportion and composition of infiltrating lymphocyte populations; and (d) correlation of *N*‐glycan pattern in TIF and corresponding serum with clinical outcome. Levels of five differentially abundant *N‐*glycans correlated with levels in TIF and matched serum (GP8, GP9, GP14, GP23, and coreF). Importantly, the prognostic potential of GP23 and coreF was validated in an independent serum cohort. These *N‐*glycans most likely reflect the signaling events underlying tumor biology and progression and may have potential for use as biomarkers to improve the diagnostic and prognostic stratification of BC. In the current study, we were not able to estimate whether particular adjuvant therapies would have had any impact on the abundance patterns of released *N*‐glycan in association with clinical outcome due to diversity of the treatment applied to the patients included in the discovery set. Further evaluation of the presented data using large independent dataset of serum from patients with breast cancer should be performed in a future.

## Author contributions

IIG and ALBD conceived and initiated this study. IIG, VDH, RS, and PMR designed the experiments. TT and EP designed the statistical and bioinformatic analyses. IIG and PSG collected samples and provided clinical information. RS and HS carried out glycan profiling. TT performed the computational analyses. MKH supervised the survival analysis. IIG, TT, and EP interpreted the results and drew the conclusions. TT with significant input from IIG, EP, PSG, and RS wrote the initial draft of the manuscript, which was reviewed by coauthors, including ÅH and OCL.

## Supporting information


**Fig. S1**. The representative images of TILs distribution within a single tumor biopsy based on the IHC analysis.

**Fig. S2.** Cox proportional‐hazard regression with known cause of death.

**Fig. S3**. The segregation of MDG BC cancer and normal serum based on the level of five *N*‐glycans groups exhibited differential abundance across TIF, NIF and matched serum.

**Table S1**. The biopsies with ≥ 1% of the invasive cancer cells positively stained for ER‐ and PgR were classified as positive.

**Table S2**. Complete characteristics of 85 breast cancer patients enrolled in the study.

**Table S3.** Glycan peaks and corresponding *N*‐glycan features.

**Table S4.** *N*‐glycans identified as differentially abundant between samples with high and low tumor TILs. *N*‐glycans are reported with associated log fold changes and adjusted *P*‐values.

**Table S5.** *N*‐glycans with significantly correlated abundances between paired TIF and serum samples. *N*‐glycans are reported with associated pearsons correlation score and adjusted *P*‐value.
