# Supplementary material for: N‐glycan signatures identified in tumor interstitial fluid and serum of breast cancer patients: association with tumor biology and clinical outcome
Source: Mol Oncol. 2018 May 14;12(6):972–90. doi: 10.1002/1878-0261.12312 (PMC5983225; doi:10.1002/1878-0261.12312)
Supplement: Supplementary file 1 — Fig. S1. The representative images of TILs distribution within a single tumor biopsy based on the IHC analysis. [file MOL2-12-972-s001.pdf]

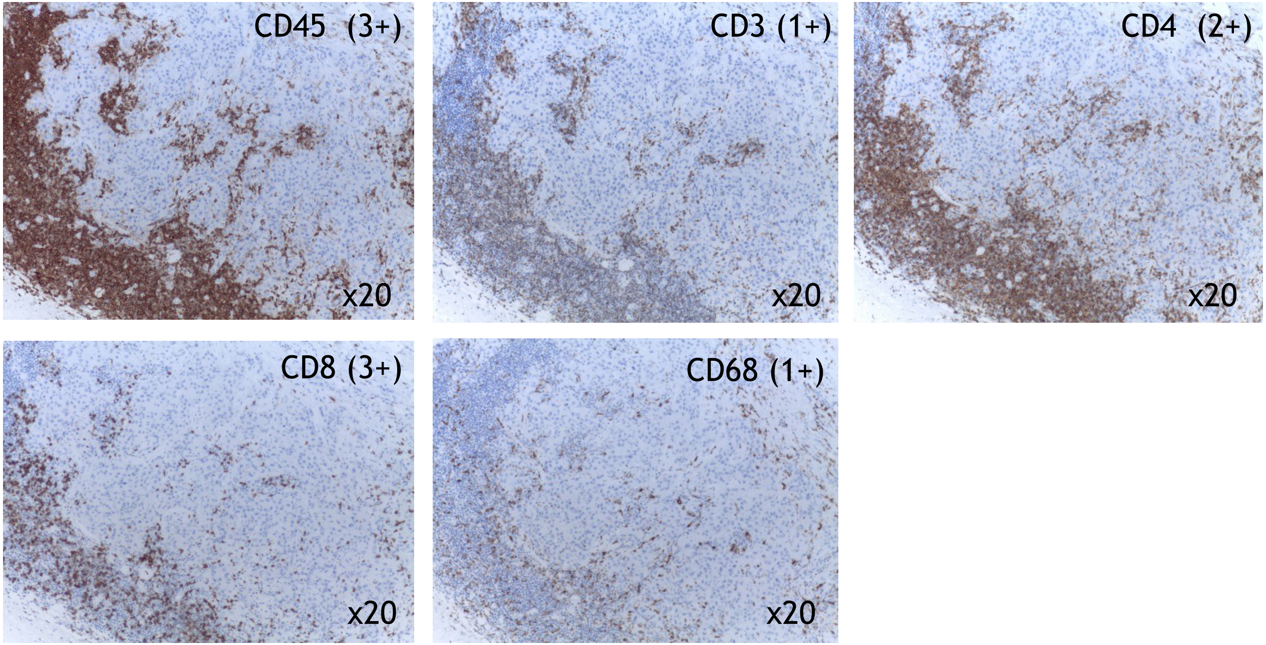

Supplementary Figure 1. The representative images of TILs distribution within a single tumor biopsy based on the IHC analysis. The representative areas for each type of TILs analyzed are shown. CD45 (the total number of infiltrating leukocytes), CD3(T cell lymphocytes of lymphoid origin), CD8 (cytotoxic T lymphocytes of lymphoid origin) and CD68 (common macrophages antigen) staining. The proportion of particular TIL subtype is scored as described in Material and Methods. Magnification shown is x20.v
