# Supplementary material for: N‐glycan signatures identified in tumor interstitial fluid and serum of breast cancer patients: association with tumor biology and clinical outcome
Source: Mol Oncol. 2018 May 14;12(6):972–90. doi: 10.1002/1878-0261.12312 (PMC5983225; doi:10.1002/1878-0261.12312)
Supplement: Supplementary file 2 — Fig. S2. Cox proportional‐hazard regression with known cause of death. [file MOL2-12-972-s002.pdf]

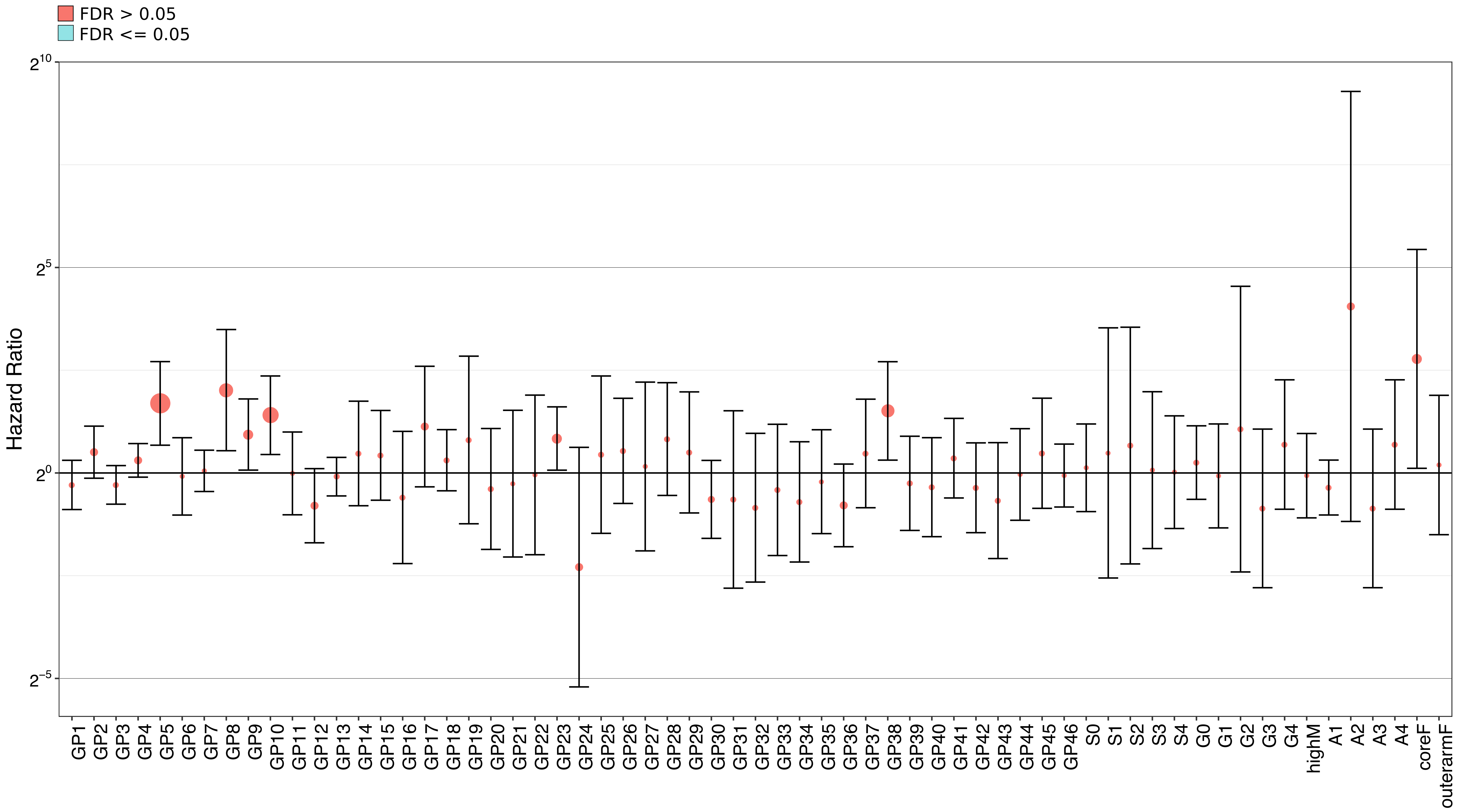

Supplementary Figure 2. Cox proportional-hazard regression with known cause of death.
Dot-plot shows hazards and confidence intervals for each N-glycan group. Dot size indicates inverse FDR, i.e., small FDRs are depicted as larger dots. Dot color indicates whether the abundance of a given N-glycan was significantly associated with patient surival (FDR <= 0.05), here events are defined as deaths were cause of death was known, with status "malignant neoplasm of breast".
