# Supplementary material for: N‐glycan signatures identified in tumor interstitial fluid and serum of breast cancer patients: association with tumor biology and clinical outcome
Source: Mol Oncol. 2018 May 14;12(6):972–90. doi: 10.1002/1878-0261.12312 (PMC5983225; doi:10.1002/1878-0261.12312)
Supplement: Supplementary file 3 — Fig. S3. The segregation of MDG BC cancer and normal serum based on the level of five N‐glycans groups exhibited differential abundance across TIF, NIF and matched serum. [file MOL2-12-972-s003.pdf]

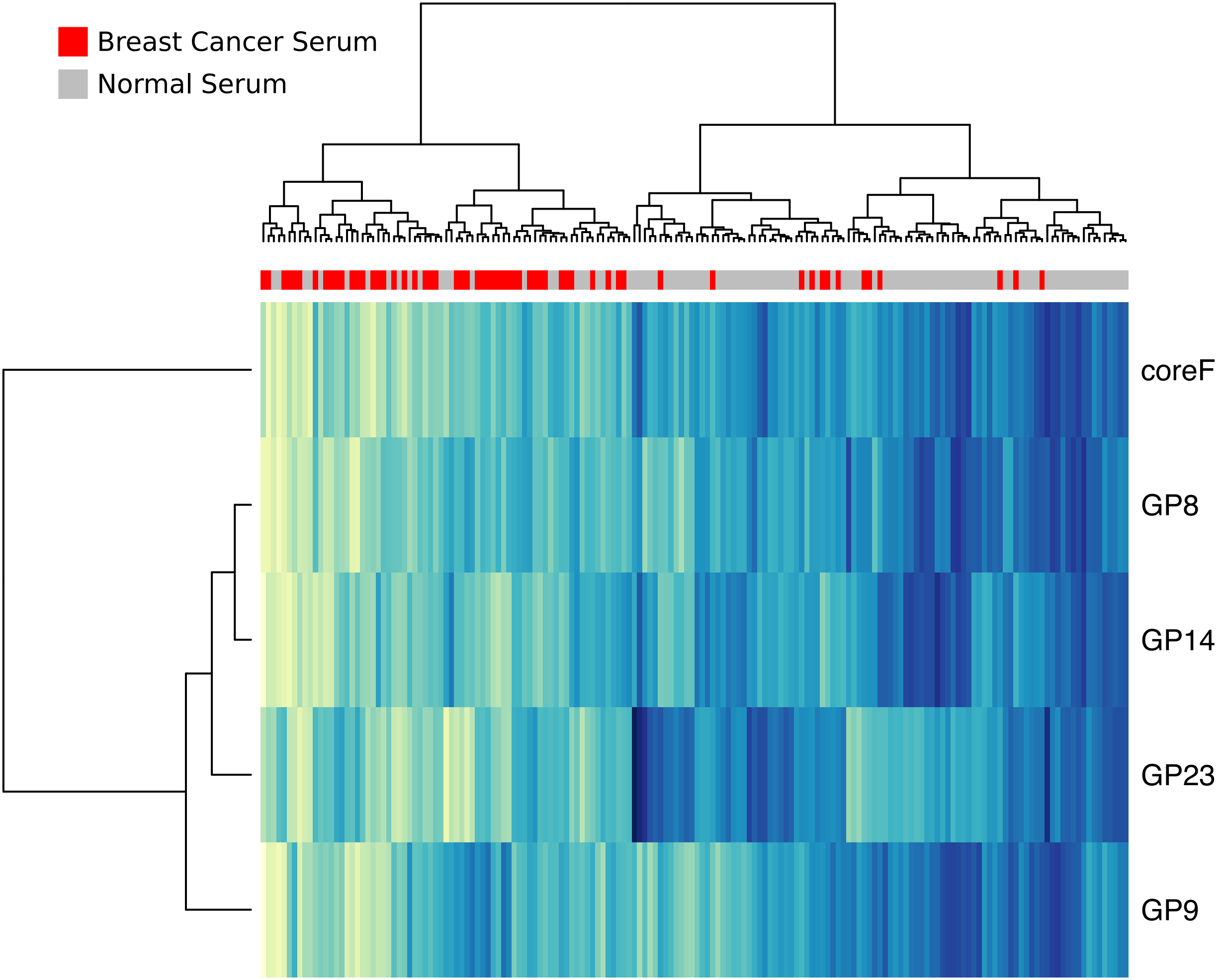

Supplementary Figure 3. The segregation of MDG BC cancer and normal serum based on the level of five N-glycans groups exhibited differential abundance across TIF, NIF and matched serum. Heatmap shows the separation of cancer and normal serum based on the abundances of five N-glycan groups found to be differentially abundant in the MDG cohort and between TIF-NIF and matched serum. Columns = samples. Colors indicate sample type. Rows = N-glycan labels
