# Supplementary material for: N‐glycan signatures identified in tumor interstitial fluid and serum of breast cancer patients: association with tumor biology and clinical outcome
Source: Mol Oncol. 2018 May 14;12(6):972–90. doi: 10.1002/1878-0261.12312 (PMC5983225; doi:10.1002/1878-0261.12312)
Supplement: Supplementary file 4 — Table S1. The biopsies with ≥ 1% of the invasive cancer cells positively stained for ER‐ and PgR were classified as positive. [file MOL2-12-972-s004.docx]

**Supplementary Table 1**: The biopsies with ≥ 1% of the invasive cancer cells positively stained for ER- and PgR were classified as positive. Tumor biopsies were considered as HER2-positive if their membrane positivity was 3+ and/or the fluorescence *in situ* hybridization (FISH) ratio of HER2 to CEP17 was ≥ 2.0. A HER2 IHC score of 2+, was also evaluated by FISH and a value < 2.0 was considered negative, while a value ≥ 2.0 was considered positive. We defined luminal B HER2-enriched tumors (LumB HER2 +) as a separate group based on the assumption that HER2 amplification along with ER or PgR positivity within luminal B subtype has an essential impact on the tumor biology. Mean Ki67 expression was used for subtype estimation and the cutoff for Ki67 positivity was assigned in accordance with currently accepted criteria (Esposito et al., 2015). Ki67 index values were measured using the open access web application, ImmunoRatio, which performs automated image analysis (Espinoza et al., 2016). * HER2+ denotes the amplification of the HER2 locus.

| **Intrinsic subtype** | **ER** | **HER2*** | **Ki67average** |
| --- | --- | --- | --- |
| luminal A | + | - | <15% |
| luminal B | + | - | ≥15% |
| luminal B HER2+ | + | + | Any |
| TNBC | - | - | Any |
| HER2+ | - | HER + | Any |
